# Predictive analytical model for ectopic pregnancy diagnosis: Statistics vs. machine learning

**DOI:** 10.3389/fmed.2022.976829

**Published:** 2022-09-23

**Authors:** Ploywarong Rueangket, Kristsanamon Rittiluechai, Akara Prayote

**Affiliations:** ^1^Department of Obstetrics and Gynecology, Phramongkutklao Hospital, Bangkok, Thailand; ^2^Department of Computer and Information Science, Faculty of Applied Science, King Mongkut’s University of Technology North Bangkok, Bangkok, Thailand

**Keywords:** ectopic pregnancy, pregnancy of unknown location, machine learning, neural networks, decision tree and support vector machines

## Abstract

**Objective:**

Ectopic pregnancy (EP) is well known for its critical maternal outcome. Early detection could make the difference between life and death in pregnancy. Our aim was to make a prompt diagnosis before the rupture occur. Thus, the predictive analytical models using both conventional statistics and machine learning (ML) methods were studied.

**Materials and methods:**

A retrospective cohort study was conducted on 407 pregnancies with unknown location (PULs): 306 PULs for internal validation and 101 PULs for external validation, randomized with a nested cross-validation technique. Using a set of 22 study features based on clinical factors, serum marker and ultrasound findings from electronic medical records, analyzing with neural networks (NNs), decision tree (DT), support vector machines (SVMs), and a statistical logistic regression (LR). Diagnostic performances were compared with the area under the curve (ROC-AUC), including sensitivity and specificity for decisional use.

**Results:**

Comparing model performance (internal validation) to predict EP, LR ranked first, with a mean ROC-AUC ± SD of 0.879 ± 0.010. In testing data (external validation), NNs ranked first, followed closely by LR, SVMs, and DT with average ROC-AUC ± SD of 0.898 ± 0.027, 0.896 ± 0.034, 0.882 ± 0.029, and 0.856 ± 0.033, respectively. For clinical aid, we report sensitivity of mean ± SD in LR: 90.20% ± 3.49%; SVM: 89.79% ± 3.66%; DT: 89.22% ± 4.53%; and NNs: 86.92% ± 3.24%, consecutively. However, specificity ± SD was ranked by NNs, followed by SVMs, LR, and DT, which were 82.02 ± 8.34%, 80.37 ± 5.15%, 79.65% ± 6.01%, and 78.97% ± 4.07%, respectively.

**Conclusion:**

Both statistics and the ML model could achieve satisfactory predictions for EP. In model learning, the highest ranked model was LR, showing that EP prediction might possess linear or causal data pattern. However, in new testing data, NNs could overcome statistics. This highlights the potency of ML in solving complicated problems with various patterns, while overcoming generalization error of data.

## Introduction

Ectopic pregnancy (EP) occurs when a fertilized egg implants outside the uterine cavity, resulting from numerous factors that interrupt the successful migration of the conceptus (1). The incidence rate of EP in Thailand and worldwide was 9.3 and 10–20 per 1,000 pregnancies, respectively (1–4). Unfortunately, the mortality rate was high compared with low numbers of incidence, since EP was reported as a major cause of maternal death in early pregnancy (5, 6). UK’s Healthcare Safety Investigation Branch (HSIB) has uncovered that failure or delay in diagnosis was the main concern (7, 8) and declared early diagnosis of EP to be a life-or-death medical decision (9).

EP is usually diagnosed in the first trimester of pregnancy. Presenting symptoms range from vaginal bleeding, abdominal pain, missed menstruation, and fainting. Examination findings include abdominal and/or adnexal tenderness, cervical motion tenderness, or hypotension. Unfortunately, many studies found that history and physical examination do not reliably predict outcomes, because up to 50% of patients revealed no risk factor (10) and 9% reported no pain. Also, the normal examination found nearly one-third of cases (11). When a pregnancy test was confirmed and early pregnancy complications were suspected, ultrasound examination was commonly used to confirm the location of pregnancy (12). However, only 73.9% of tubal EPs were visualized by initial TVS (13), and those with no signs of intra- or extrauterine pregnancy on transvaginal ultrasonography would initially be defined as pregnancy of unknown location (PUL), ranging from 8 to 31% in prevalence. Within this cohort, one-third was found as early intrauterine pregnancy, and a range of 8.7–42.8% was found as EP (14–16). Recently, serial measurements of serum hCG have been shown to improve the diagnostic rate (12). Unfortunately, the result could not differentiate those with EP from normal intrauterine pregnancy or miscarriage precisely enough (17). Consequently, clinicians misdiagnosed more than 40% of EPs on the initial ED visit reported in a former study (18). While many clinical protocols have been improved, in addition to modern investigation tools, limitations remain in diagnosis as observed in the UK. The National Health Service (NHS) uncovered 30 missed EPs leading to “serious harm” in 1 year (2017–2018) (9). Therefore, this time-critical condition can become life-threatening when the implantation site ruptures causing immediate bleeding into the intra-abdomen and eventually leading to hemorrhagic shock.

Attempts in developing models for EP diagnosis were established from a variety of domains. These include the clinical risk factor model, classifying elevated risk group (19) or the risk factor model combined with a single serum hCG (20). While the specificity was high, the sensitivity was inconsistent. Second, the serum level of the progesterone model with single cutoffs had AUC 0.725, or the later widely known model using serial hCG, M1, M4, and M6, was presented with high performance. However, the accuracy was lower in different cohorts and required at least two follow-up examinations up to 48 h (21–23). Finally, the ultrasound score classified patients using ultrasound findings (24), and there were still unavoidable limitations of ultrasound user expertise and patient’s confounding factors.

In addition to the limitations mentioned, international consensus remains lacking, with no gold standard tools have been established to identify early EP. Our aim was to develop a model combining all three domains using traditional statistical analysis and machine learning (ML).

ML has dramatically contributed to new knowledge in the medical field in the last two decades. It has defined the evolution of interdisciplinary sciences between statistics, artificial intelligence, and medicine. It possesses the ability to conduct complex tasks, automatically, determines hidden patterns that are too complex for humans to observe, and has the advantage of discovering rules for behavior and adaption to changes in wording, making ML suitable to predict new EP cases (25).

Numerous EP studies have been based on traditional statistical analysis. Although EP is dangerous and difficult to detect, small numbers of studies have applied ML in this field. One was used as a decision support model for treatment. Interestingly, another studied different ML methods to predict EP in PUL based on serum hCG and clinical information. To the best of our knowledge, this is the first study to combine all diagnostic feature domains using both statistics and ML methods.

Our research problem applied the classification technique based on a supervised learning method. The widely used ML methods include the decision tree (DT), support vector machine (SVM), neural network (NN), and logistic regression (LR), which is the traditional and most used statistical method. Each model process uses distinctive characteristics of algorithms that are suitable for different sets of data problems. Our study aimed to compare all four models and determine the best model suitable for the stated problem.

## Materials and methods

### Problem definition and formulation

**FIGURE 1:**
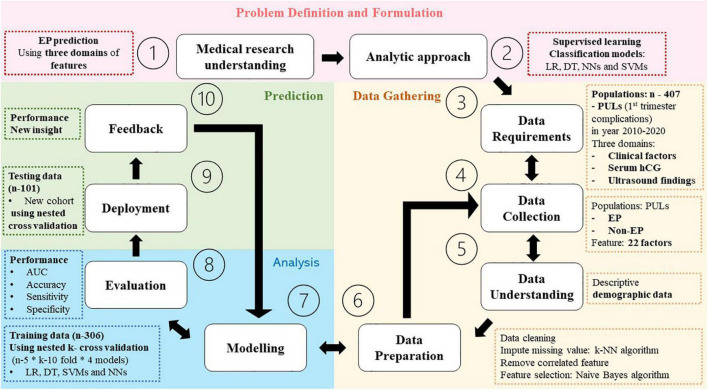
Study flow diagram based on the foundational method for data science (FMD), IBM (27, 28).

This constituted a retrospective cohort study, briefly summarized in Figure 1, conducted from electronic medical records of 1,604 pregnant women presenting first trimester complication symptoms including abdominal pain and/or abnormal vaginal bleeding at Phramongkutklao Hospital between October 2010 and March 2022. The criteria for inclusion were those suspected of PUL with medical report of clinical history, physical examination, and ultrasound evaluation. Women were included regardless of the report with or without taking serum hCG due to medical judgment at that time, such as those presenting suspicious signs of intrauterine pregnancy or extrauterine pregnancy *via* ultrasonography at the first visit. The patients presenting clinically suspicious ruptured EP (clinical instability or sign of intra-abdominal hemorrhage) or showing any evidence of intrauterine gestational content or EP (adnexal mass consisting of fetal pole or fetal heart motion) by ultrasound at the first visit were excluded. EPs were those diagnosed with pathological reports in surgical cases and abnormal serial serum hCG in non-surgical cases. The study was approved by the Royal Thai Army Medical Department Institutional Review Board, reference number R048h/62_Exp. Patient identification was coded before analysis and discussion. We declare that we used some parts of identical electronic medical data of patients, visiting Phramonkutklao Hospital, for model validation in this research, using different research questions and methods (26).

#### Outcome of measurement

Binomial value (ectopic pregnancy/non-ectopic pregnancy).

#### Analysis

**FIGURE 2:**
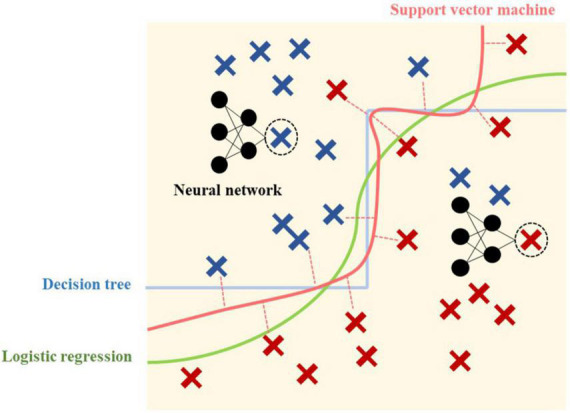
Conceptual overview of four predictive models.

For supervised learning analysis, four basic and powerful classification methods were chosen for their unique classifying ability as conceptually demonstrated in Figure 2. Despite the development of a variety of methods, each method provides its own characteristics, and the method capability and model requirement should be matched.

#### Logistic regression

Is a traditional statistical method, invented by a British statistician David Cox in 1958 (29), dealing with classification problems using a logistic function, for which the result always falls between 0 and 1 and the graph of the function is S-shaped. The regression method has an advantage in its interpretability, which could explain how the model works and more importantly lead to an understanding of “why?” this patient was predicted “yes”. Although regression coefficients in LR are challenging to interpret and understand as in linear regression, it could interpret whether the relationship is proportional or inversely proportional between each feature (probability) (30, 31).

#### Support vector machines

Are also used for classification as an alternative to LR, devised by Soviet statisticians in 1963, and have become feasible with the introduction of kernels and soft margin classifiers in the 1990s (32). The advantage over simple regression is that linear or LR uses all the data points in the calculation of the line of best fit, while SVMs focus on only the set of points (called “support vector”) closest to the margin. However, in terms of interpretability, SVMs perform relatively like a black box (31).

#### Decision trees

Classify training data by sorting them on a tree from root to leaf nodes downwardly. Each internal node involves a feature and prediction made at leaf nodes. A leaf is a collection of examples that may not be classified any further (33). It has the ability to sequence both discrete and continuous values and can be used even when some training data have unknown values (33). However, practical issues arise from learning to determine how deeply to grow the DT, manage continuous data, choose an appropriate feature selection measure, and link training data with missing attribute values.

#### Neural networks

Were named as a simulation of how brain cellular networks function, which were used in the 1950s. NN comprises one or more layers of autonomous computational units or nodes receiving input from other nodes (including within the same layer), and sending output or even feedback to previous input, to present the final output prediction. Although the earliest NNs were used in classification prediction like basic SVMs or linear discriminant analysis, they have become more useful in more complex or non-linear tasks like handwriting or imaging recognition, which are competitive in solving real-world problems, including using non-linear data. However, one disadvantage found in NN was the longer time required for model running compared with the same category of problems by LR, SVM, and DT. Second, only numbers of nodes and layers were identified. Finally, NNs have no explanatory power to explain “why” this is predicted.

#### Software

RapidMiner Studio 9.9.003 is a well-known data analyst tool, especially used for predictive analysis and statistical computing (34, 35).

### Data gathering

#### Study population

In total, 407 PUL patients (26) were included in this study.

#### Features (predictive values)

Three domains of 22 features of categorical data comprise clinical history (demographic data, risk factor history, clinical manifestations), initial serum hCG levels, and ultrasound results. All factors were extracted and selected from literature reviews that were statistical and clinically relevant to our research outcome.

#### Data preparation

##### Missing value

Due to the nature of a retrospective study, missing input data are inevitable. After reviewing cases, approximately 10–20% involved missing values in all 22 features and were missing at random (mostly insignificant negative findings or assumed irrelevant history in those hospital visits) (36). Our objective was to understand the data for training, not deleting ones, which could bias the classifier performance (37). The missing value imputation method has been shown to improve prediction capability. Thus, the Naïve-Bayes, a simple, probability ML, was applied (38).

##### Remove correlated features

To avoid confusing correlation and causation, features with high or substantial absolute correlation of more than 0.95 were removed (39).

##### Feature selection

To select the attribute that was most useful for classifying examples, optimization selection using forward/backward stepwise was applied [*n* (generation without improvement) = 1].

### Model analysis

#### Dataset allocation

To maximize the use of all values, while decreasing generalization error or testing/training dataset variance, the nested cross-validation technique (40) was applied by randomly splitting and selecting a training and testing dataset (306:101) in five different loops. In addition, an inner loop 10-fold cross-validation of training/validating data was added. The performance would present in average, minimum, and maximum values from all five-model loops’ analyses.

#### Model training and validating (internal validation/*n* = 306)

To optimize the process of training sets to estimate their accuracy and to overcome model overfitting, by providing 10-fold (9:1) training and validating data (25), all four models were trained using the entire dataset.

#### Performance evaluation

Comparing the four models, the area under the receiver operating characteristic curve (ROC-AUC) (41) was used to report the mean ± SD of the cross-validation process. Also, report accuracy, sensitivity, specificity, positive predictive value (PPV), and negative predictive value (NPV) were employed to gain more insights.

### Prediction

#### Model testing/deployment (external validation/*n* = 101)

All four models were applied to newly separated patients’ data using the nested cross-validation technique, and then, the model performance was compared using ROC-AUC.

## Results

### Characteristics of study populations

Of the 1,604 pregnant women with first trimester complications, 407 (25.4%) patients initial visits were identified as PUL—the final diagnosis totaled 208 (51.1%) EPs and 199 (48.9%) non-EPs. Among non-EPs, 22 (11.1%) were threatened abortion, 1 (0.5%) blighted ovum, 1 (0.5%) corpus luteal leakage, and the other 175 (87.9%) constituted spontaneous abortion. The mean age was 30 years with 55.3% multiparity. Comparing the demographic data presented in Table 1, no difference was found between the two groups.

**TABLE 1:** Descriptive demographics and features of the study population.

	Characteristics	Total (*N* = 407)	*N* (%)	EP *N* (%) (*N* = 208)		Non-EP (*N* = 199)	*N* (%)	*P*-value
**Demographic data: Age (years) mean ± SD**				29.58 ± 6.08		30.86 ± 6.30		
	**Age group (years)**							
	<35 (%)	304	(74.69%)	161	(52.96%)	143	(47.04%)	0.198^a^
	≥35 (%)	103	(25.31%)	47	(45.63%)	56	(54.37%)	
	**BMI (kg/m^2^)**							
	<23 (kg/m^2^) (%)	272	(66.83%)	141	(51.84%)	131	(48.16%)	0.675^a^
	≥23 (kg/m^2^) (%)	135	(33.17%)	67	(49.63%)	68	(50.37%)	
	**Parity**							
	Nulliparity (%)	182	(44.72%)	85	(46.70%)	97	(53.30%)	0.110^a^
	Multiparity (%)	225	(55.28%)	123	(54.67%)	102	(45.33%)	
	**Gestational age at diagnosis (days)**			52.40 ± 15.78		51.82 ± 16.43		0.717^b^
**Risk factors: History of pelvic surgery**								
	No (%)	304	(78.15%)	164	(53.95%)	140	(46.05%)	0.351^a^
	Yes (%)	85	(21.85%)	41	(48.24%)	44	(51.76%)	
	**Smoking**							
	No (%)	380	(97.94%)	198	(52.11%)	182	(47.89%)	0.007^a^
	Yes (%)	8	(2.06%)	8	(100.00%)	0	(0.00%)	
	**History of ectopic pregnancy**							
	No (%)	385	(95.77%)	197	(51.17%)	188	(48.83%)	0.886^a^
	Yes (%)	17	(4.23%)	9	(52.94%)	8	(47.06%)	
	**History of pelvic inflammatory disease (PID)**							
	No (%)	347	(96.12%)	188	(54.18%)	159	(45.82%)	0.020^a^
	Yes (%)	14	(3.88%)	12	(85.71%)	2	(14.29%)	
	**Current use of emergency pill**							
	No (%)	331	(89.46%)	162	(48.94%)	169	(51.06%)	<0.001^a^
	Yes (%)	39	(10.54%)	33	(84.62%)	6	(15.38%)	
	**Assisted reproductive technology**							
	No (%)	390	(97.26%)	200	(51.28%)	190	(48.72%)	0.831^a^
	Yes (%)	11	(2.74%)	6	(54.55%)	5	(45.45%)	
**Symptoms: Abdominal pain**								
	No (%)	79	(19.46%)	27	(34.18%)	52	(65.82%)	0.001^a^
	Yes (%)	327	(80.54%)	180	(55.05%)	147	(44.95%)	
	**Abnormal vaginal bleeding**							
	No (%)	84	(20.64%)	51	(60.71%)	33	(39.29%)	0.048^a^
	Yes (%)	323	(79.36%)	157	(48.61%)	166	(51.39%)	
	**Nausea, vomiting**							
	No (%)	267	(85.85%)	146	(54.68%)	121	(45.32%)	0.408^a^
	Yes (%)	44	(14.15%)	27	(61.36%)	17	(38.64%)	
	**Fainting**							
	No (%)	293	(92.43%)	156	(53.24%)	137	(46.76%)	0.014^a^
	Yes (%)	24	(7.57%)	19	(79.17%)	5	(20.83%)	
**Signs**	**Abdominal tenderness**							
	No (%)	209	(51.35%)	67	(32.06%)	142	(67.94%)	<0.001^a^
	Yes (%)	198	(48.65%)	141	(71.21%)	57	(28.79%)	
	**Cervical motion tenderness**							
	No (%)	324	(80.19%)	126	(38.89%)	198	(61.11%)	<0.001^a^
	Yes (%)	80	(19.81%)	79	(98.75%)	1	(1.25%)	
**Serum marker: Serum β -hCG level at first visit (mIU/ml)**								
	<1,000 (%)	153	(53.87%)	46	(30.07%)	107	(69.93%)	<0.001^a^
	≥1,000 (%)	131	(46.13%)	74	(56.49%)	57	(43.51%)	
**Ultrasound findings: Intra-uterine anechoic content**								
	No (%)	351	(86.88%)	182	(51.85%)	169	(48.15%)	0.251^a^
	Yes (%)	53	(13.12%)	23	(43.40%)	30	(56.60%)	
	**Endometrial thickness > 14mm**							
	No (%)	321	(83.38%)	163	(50.78%)	158	(49.22%)	0.304^a^
	Yes (%)	64	(16.62%)	28	(43.75%)	36	(56.25%)	
	**Adnexal mass of complex echogenicity**							
	No (%)	191	(47.04%)	26	(13.61%)	165	(86.39%)	<0.001^a^
	Yes (%)	215	(52.96%)	181	(84.19%)	34	(15.81%)	
	**Free fluid in cul-de-sac**							
	No (%)	235	(58.75%)	74	(31.49%)	161	(68.51%)	<0.001^a^
	Yes (%)	165	(41.25%)	129	(78.18%)	36	(21.82%)	

SD, Standard deviation; BMI, body mass index (kg/m^2^); PID, pelvic inflammatory disease.

^a^Chi-square test. ^b^*t*-test.

**FIGURE 3:**
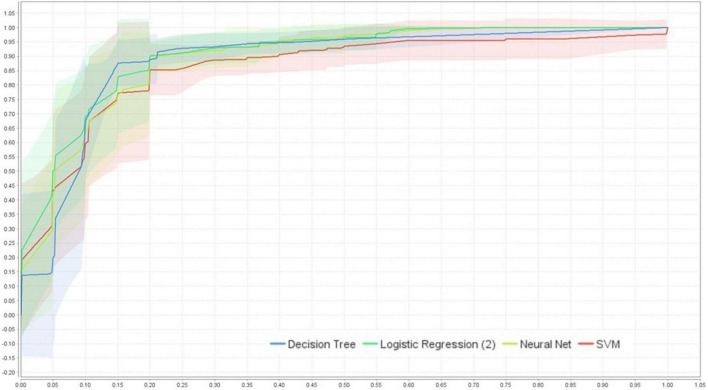
ROC-AUC (95%CI) performance comparison of the four models using cross-validation (internal validation), created by RapidMiner Studio 9.9.003.

Regarding the data preparation process, features selected to be in the model are shown in Table 2. We then ran the model analysis and presented the performance comparison in ROC-AUC in Figure 3.

**TABLE 2:** Features selected in the four models.

Machine learning model	Feature selections (yes/no)
Logistic regression, support vector machine	Multipara, vaginal bleeding, cervical tenderness, serum hCG ≥ 1,000 mIU/mL, inhomogeneous adnexal mass in ultrasound
Neural network	Multipara, history of pelvic surgery, cervical tenderness, serum hCG ≥ 1,000 mIU/mL, inhomogeneous adnexal mass, intrauterine anechoic sac in ultrasound
Decision tree	History of pelvic inflammatory disease, emergency pill, nausea-vomiting, cervical tenderness, serum hCG, inhomogeneous adnexal mass, free fluid in ultrasound

hCG, human chorionic gonadotropin.

**FIGURE 4:**
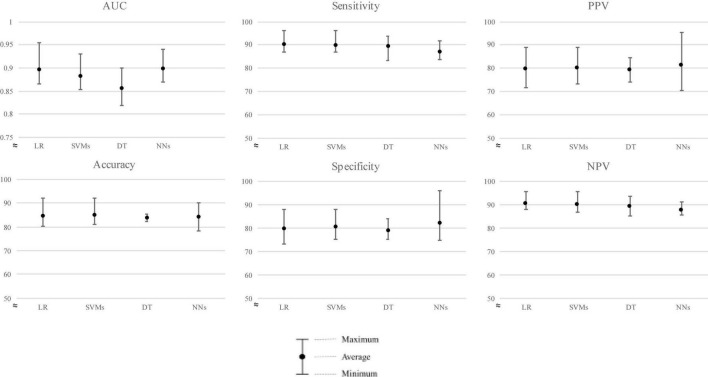
Predictive performance of the four models (external validation).

The average performance ROC-AUC was high in all models (AUC ≥ 0.856, Figures 3, 4), also highlighting that the statistical model (LR) was superior to ML in training validation, while NNs were more superior in external testing.

Of the four models’ performances in the testing population shown in Figure 4, NNs ranked first, followed closely by LR, SVMs, and DT with average ROC-AUC ± SD of 0.898 ± 0.027, 0.896 ± 0.034, 0.882 ± 0.029, and 0.856 ± 0.033, respectively. For clinical aid, we reported sensitivity of mean (± SD) LR: 90.20% ± 3.49%, SVM: 89.79% ± 3.66%, DT: 89.22% ± 4.53%, and NNs: 86.92% ± 3.24%, respectively. Furthermore, specificity of mean (± SD) was ranked by NNs, followed by SVMs, LR, and DT, i.e., 82.02 ± 8.34%, 80.37 ± 5.15%, 79.65% ± 6.01%, and 78.97% ± 4.07%, respectively.

**FIGURE 5:**
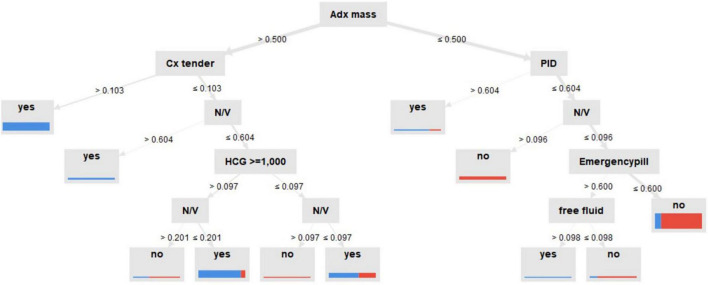
Decision tree model for predictive ectopic pregnancy diagnosis. Adx mass: inhomogeneous adnexal mass, N/V: nausea-vomiting, Cx tender: cervical tenderness, PID: pelvic inflammatory disease, created by RapidMiner Studio 9.9.003.

Figure 5 shows more insight on how the DT model predicts the outcome, indicating that the prediction process was in a prioritizing order. As the tree grows downward, we found that the adnexal mass was the highest of priority decision nodes, first used to classify patients, indicating it as the main classified feature, followed by cervical tenderness. Concerning the second pathway if none of these two features existed, we found that the initial serum history of PID, nausea-vomiting symptoms, and current use of emergency pill could provide additional decisional data, as well as serum hCG of more than 1,000 mIU/mL in another branch.

We then evaluated the models on the new cohort for external validation and found that NNs models performed best with ROC-AUC of 0.898, followed by LR, SVMs, and DT as shown in Table 3. However, a slight difference in performance was observed between LR and NNs in both internal and external validations.

**TABLE 3:** Average ROC-AUC performance comparison of the four models applied to the internal and external validation datasets.

Model	Internal validation		External validation	
	Average	S.D.	AUC	S.D.
LR	0.879	0.010	0.896	0.034
SVMs	0.869	0.016	0.882	0.029
DT	0.855	0.009	0.856	0.033
NNs	0.876	0.012	0.898	0.027

AUC, area under the curve; S.D., standard deviation.

## Discussion

Our study reported an incidence of 51.1% EPs among initially suspected women with PUL. However, other studies reported an incidence ranging from 7 to 31% (14, 15, 42, 43). A similar rate was observed in one large prospective observational study by Malek-mellouli et al. (44) with a rate of 43%. This could be explained by spontaneous resolution of EP in PUL that might constitute a failed diagnosis, because the location remained unknown, while some cases might have been misclassified as missed abortion. Also, the higher number might have resulted from the sensitivity of ultrasound at the initial diagnosis that subjectively differed between cohorts. Gestational age of diagnosis was also similar in both groups, leading to a challenge for early diagnosis. Using the method of data science for model development, two main steps of results were developed.

### First, model learning and validation

While ML is believed to empower prediction fields, theoretically using complex algorithms should enable highly accurate models (45). Our result showed that LR provided a better predictive ability throughout ROC-AUC. One explanation could be that the model was obtained from features, chosen by reviewing and studying many literature reviews, resulting in true causation, which was unavoidable because medical data are reasonably based on fact. Evidently, due to the presence of non-random variation (causal or linear relationship) in the input variables, LR performed the best of the four models in the internal validation process. Interestingly, SVMs also presented the best accuracy, supporting the fact that medical data might possess a linear character, and the support vector of the SVM model exhibited a greater fit with the data (31).

Particularly interesting for researchers is the new feature, serum hCG cutoff at ≥ 1,000 mL/mL, for predicting EP. Also, another study found similar associations (46). To the best of our knowledge, no model has used this serum cutoff as a feature in prediction, yet. Also, it has been shown in these four models that cervical tenderness, adnexal mass, and serum hCG were chosen using the optimized selection feature process. This could be interpreted by the DT model. While ultrasound findings of inhomogeneous adnexal mass were prioritized, followed by physical examination of cervical tenderness, the initial level of serum hCG up to 1,000 IU/mL, clinical risk factors of nausea-vomiting, and current use of emergency pills were shown to be useful to classify women with PUL in consecutive order. This was related to the evidence based on observational studies in that these factors were shown to correlate with EP with high odds ratio (44, 47). This research might prove that the four factors were not just related to EP but might offer biological plausibility as well.

Comparing the ML models, the major drawback of ML models, especially NNs and DT, occurs in their training phase. We found that accuracy was highly dependent on the size of input data (48). Although the extraordinary generalization capability of NNs and its discriminative power make NNs perform better than DT, those models practically and theoretically achieve less than NNs. However, DT has advantages in dealing with training data missing values, which could be more useful in practical use.

### Second, model deployment, and testing

When deploying the models to unseen data, the average ROC-AUC was slightly higher in all four models, proving the generalizability of the models by defeating model overfitting. While NNs proved to be more superior in classifying EPs, followed closely by LR, highlighting the fact that EPs prediction might tend to be in linear or causal relationships due to medical data based on fact, for which LR and SVMs proved their capabilities. More importantly, NNs, which were mostly studied well using non-linear data, also proved the potential performance in this prediction. Unfortunately, due to the small sample size, further study is required for more validation. Importantly, introducing the nested cross-validation technique by randomly splitting and matching between training and testing data over five different outer loops for model evaluations provided an acceptable validation. However, when more new data become available, and more features are explored, the model could become more complex and harder to incorporate all data in a single optimal model, which could constitute a drawback of ML.

For decision-making, ideally, we would prefer a diagnostic test offering both 100% sensitivity and 100% specificity. Unfortunately, this rarely occurs and is usually viewed as a trade-off (49). In practical use, we would like to focus on two circumstances.

Concerning the first patient visit, the model focusing on EP screening might represent the most important because limitations or pitfalls occur in many settings involving the lack of obvious clinical presentations or ultrasound findings and the lack of a specialist to consult in primary care hospitals (50). To decrease the misdiagnosis rate (ruled in), high sensitivity remains crucial, for which we found LR performed the best. This was because we were concerned whether a positive disease (EP) was not identified using a positive test result (51), leading to inappropriate discharge or inadequate follow-up. We also found the least false negative case in the LR model. Furthermore, to emphasize confidence in test sensitivity, patients predicted as non-EP still need counseling to use NPV rate, because sensitivity cannot be used to categorize other people as not having the condition when in fact they do have it (for which LR also ranked first in NPV performance).

In the second circumstance, following up the EP group or, in practice, elevated risk PUL, serial serum hCG, and ultrasound would have been followed as a standard protocol to definite cases of EP and intrauterine pregnancy identified by ultrasound. Unfortunately, we found that in counseling for treatment (ruled out), high specificity was more important. As a result, NNs might be chosen, because they could perform at the best specificity. Thus, people with a positive test result would be very unlikely to be categorized as having a condition if they indeed did not have it and prevent harmful unnecessary treatment for normal pregnancy.

Therefore, selecting a model for scientific problems can markedly influence predictive performance. Building complex models using some data might create the only model that is sufficiently powerful to predict ones but might become useless concerning some questions. This is because the more complex the model, the harder the results of a prediction would be to explain, so you might never obtain the answer for “why” this says “yes.” Second, while keeping up with the changing patient’s information in the real world, simple models tend to maintain their performance, but complex ones require up-to-date maintenance. Therefore, an additional key could be to focus on the nature of the data instead of creating complex models. Finally and more importantly, decisions for what model would constitute the best might depend on the nature of data and the question of “what is the answer” vs. “why is this the answer.”

## Conclusion

Our research highlights the advantage of applying ML in medical settings as an innovative way for disease prediction using its complex algorithms to discover unknown patterns or information inside the black box. The abilities in dealing with missing values, selecting the most optimized features, and analyzing non-parametric data have proved to be ground-breaking methods for clinical use.

Study limitation was mainly due to the low incidence of EP in Thai populations. Thus, a retrospective study was chosen. However, it provided sufficient power of data for statistics, but obtained unavoidable missing data. Second, while input data type was related to the analyzing process, the prediction performance was affected by the type of data. Our research mostly used category instead of continuous data, which could have limited the performance of NNs and SVMs by its nature.

Furthermore, as healthcare organizations have produced and recorded tons of patient information, which might never be used, organized electronic collection of data could be properly processed as a medical alerting system to predict using an ML-based algorithm. With the ML model, knowledge from these data could produce an ultimate benefit in terms of predicting and inventing new insights, gaining more benefit from the experiences of previous ones (52).

## Data availability statement

The original contributions presented in this study are included in the article/supplementary material, further inquiries can be directed to the corresponding author/s.

## Ethics statement

Written informed consent for participation was not required for this study in accordance with the national legislation and the institutional requirements due to retrospective study.

## Author contributions

PR collected the data and performed the data analysis. PR and AP interpreted the data, drafted, and revised the manuscript. PR and KR approved the version of the manuscript to be published. All authors contributed to the conception and design of the study.

## Abbreviations

EP: ectopic pregnancy
PUL: pregnancy of unknown location
ML: machine learning
NNs: neural networks
DT: decision tree
LR: logistic regression
SVM: support vector machines.
